# Microplastics and gut microbiomes impact on Yunnan snub-nosed monkeys in the Three Parallel Rivers region in China

**DOI:** 10.3389/fmicb.2024.1449522

**Published:** 2024-08-12

**Authors:** Wancai Xia, Jiajie Zhou, Zhongwei Lu, Liang Li, Yuan Zhang, Shiyuan Fan, Ali Krzton, Dayong Li

**Affiliations:** ^1^Key Laboratory of Southwest China Wildlife Resources Conservation (Ministry of Education), China West Normal University, Nanchong, China; ^2^Liziping Giant Panda’s Ecology and Conservation Observation and Research Station of Sichuan Province, Science and Technology Department of Sichuan Province, Chengdu, China; ^3^Auburn University Libraries, Auburn University, Auburn, AL, United States

**Keywords:** microplastics, Yunnan snub-nosed monkey, gut microbiomes, food provisioning, tourism

## Abstract

**Background:**

Microplastics (MPs) has been rapidly increasing and interacting with wildlife. As the highest altitudes inhabited non-human primate, Yunnan snub-nosed monkey (*Rhinopithecus bieti*) have been proven to be an umbrella and flagship species to indicate ecosystem changes and help develop environmental management strategies. In this study, we aimed to investigate the behavioral and ecological reasons for the types, content and differences of MPs in the feces of *R. bieti*, and explored the effects of MPs on gut microbiome of *R. bieti*.

**Methods:**

We used the Agilent 8700 LDIR to identify the abundance and size distribution of MPs in fecal samples, and then analyzed the causes of differences in MPs content by combining data from different populations (wild group, provisioned wild group) and dominance hierarchy. At the same times, the relationships were investigated between gut microbiome diversity and MPs content.

**Results:**

We first demonstrate MPs ingestion by *R. bieti*, which highlights the potential impacts of MPs pollution in such high-altitude, inaccessible protected areas. A total of 36 types of MPs were detected, with an average of 75.263 ± 58.141MPs/g. Food provisioning and tourism significantly increased the content of MPs in the feces of *R. bieti*, but tourism alone did not significantly increase the content of MPs as food provisioning. At the same time, the study found that there was no significant difference in the content of MPs between different sex groups, however, the feces MPs content of adult *R. bieti* was significantly lower than that of juvenile, and the social dominance hierarchies among OMUs was positively correlated with the exposure of MPs. The current level of MPs pollution did not cause gut microbiome dysbiosis of *R. bieti*.

**Conclusion:**

Our study proved from behavioral and ecological perspectives that the *R. bieti* exposure to MPs was related to provisioned food, and was closely related to dominance hierarchy and age. From the perspective of intestinal microbiology, it was proved that the current intake of MPs did not cause gut microbiome dysbiosis of *R. bieti*. Our study provided scientific basis for formulating effective protection measures and promoting the effective protection of rare and endangered animals.

## 1 Introduction

Plastics have become some of the most widely used materials in industry and activities of daily life due to their light weight, durability, and low cost (Wang et al., 2023). Over the past century, global plastic production has grown exponentially (Schwabl et al., 2019), with more than 350 million tons produced annually (Geyer et al., 2017; Prata, 2017). Approximately 79% of all extant plastic has accumulated in landfills and the natural environment (Geyer et al., 2017). Microplastics (MPs) pollution has become a hot issue of global concern (Sutherland et al., 2010). In recent years, the classification, distribution, ingestion, hazard and degradation of MPs have been the research hotspots in ecology, but these studies have mainly focused on aquatic ecosystems and experimental animals. Although MPs pollution on land is 4–23 times higher than in the ocean (Horton et al., 2017), terrestrial ecosystems have received much less attention than aquatic systems. Since plastic is mostly produced, used and discarded on land (Jambeck et al., 2015), MP first interact with terrestrial ecosystems, altering their chemical and biological environments and causing environmental toxicity (Emenike et al., 2023). As a result of physical, chemical, and biological processes, plastic in the environment degrades and disintegrates to form MPs, or fragments under 5 mm long (Barnes et al., 2009; Zhang et al., 2021). These are then spread through dispersal by wind, rainfall, flowing water, the food chain, and other assorted processes (Huang et al., 2022). Eventually, they make their way into the atmosphere, soil, water, glaciers, and other inorganic environments (Koelmans et al., 2022). MPs have been detected in aquatic and terrestrial biota, as well as in humans (Prata, 2023); they are not only a source of environmental pollution, but also hazardous to organisms as a vector for contaminants such as plastic additives and microbes (Avio et al., 2015; Schwabl et al., 2019; Liu M. et al., 2023; Liu X. et al., 2023).

At present, some progress has been made in the research on the transport, concentration and degradation of MPs in the environment, the biological hazard of MPs on the body, and the combined pollution caused by the interaction of MPs with other organic pollutants and heavy metals (Jambeck et al., 2015). The previous research work has a good guiding significance for understanding and controlling MPs pollution. However, it is worth noting that at present, a large number of studies stay on the high concentration of MPs in laboratory animals to explore the hazard to animals, while there are few studies on wild animals.

After plastic are discharged into the environment, plastic particles with a diameter of less than 5 mm are called MPs through physical and chemical interactions. MPs in the environment can be divided into primary MPs and secondary MPs. Plastics that are fabricated directly into microscopic dimensions are defined as primary MPs. Primary MPs are mainly derived from cosmetics, air blasting media, drug carriers, and primary plastic production particles. About 79% of plastics are exposed to terrestrial ecosystems (Geyer et al., 2017) and are broken down into secondary MPs through degradation by mechanical, chemical and biological processes (Zhang et al., 2021). Therefore, the sources of secondary MPs are diverse, such as degradation, aging, oxidation, and wear, and it is difficult to determine the exact source of secondary MPs at present. Primary and secondary MPs are transmitted through routes such as wind, rain and running water (Huang et al., 2022) and enter inorganic environments such as atmosphere, soil, water and glaciers (Koelmans et al., 2022).

Although there are relatively few studies on plastic pollution in terrestrial vertebrates, some insects, reptiles, birds and mammals have been shown to ingests microplastics (Robson et al., 2021), and the ingestion of MPs by animals is mainly through food, water and air (Huang et al., 2021). Due to the influence of transport media, the transport mode of plastics in terrestrial and aquatic environments is different (Liu X. et al., 2023), and most of the MPs in terrestrial ecosystems are regionally concentration. Many studies have also proved that the difference in MPs content in different geographical populations is closely related to the regional concentration of microplastics (Ding et al., 2023). The transport of MPs overlap with the food chain (Robson et al., 2021). In addition to the ecological transport pathway, we hypothesize that MPs transport is also closely related to animal behaviors. The redistribution of MPs concentration food resources can be determined by social behaviors to a certain extent. For example, the resource preference hypothesis proposes that high dominance hierarchies individual preferentially occupy the precious resources (Tibbetts et al., 2022). Juvenile preferred to interact with tourists (Xia et al., 2016), which increased the risk of MPs ingestion. The MPs content of *Gadus chalcogrammus* was positively correlated with age (Ding et al., 2023).

MPs ingestion is one of the reasons for the dysbiosis of host gut microbiome, because MPs ingestion is accompanied by a range of factors known to drive gut dysbiosis, such as malnutrition, inflammation, introduction of pathogens, endocrine disruptors, and environmental chemicals (Fackelmann and Sommer, 2019). At present, it is intuitively reflected in the change of the diversity of gut microbiome, leading to significant shifts in the relative abundances of certain phyla (Bacteroidetes, Firmicutes, and Proteobacteria), which are common in gut microbiome studies (Jin et al., 2018; Lu et al., 2018; Zhu et al., 2018; Qiao et al., 2019). The results of common laboratory studies almost all prove that MPs exposure alters the diversity of gut microbiome, resulting in gut microbiome communities distinct from the controls without MPs treatments (Fackelmann and Sommer, 2019). How significant is the difference in gut microbiome caused by the huge difference in MPs content between treatment and control groups in laboratory conditions to guide wildlife?

The Yunnan snub-nosed monkey (*Rhinopithecus bieti*) is a rare and endangered primate unique to China, and they inhabit the alpine primary forest in the area of the Three-river parallel flow area (Xia et al., 2020). Due to the impact of human activities such as environmental pollution and tourism development, MPs inevitably invade their habitats. As an important flagship species with only about 3,000, *R. bieti* is very sensitive to environmental pollution (Xia et al., 2020). In view of the hazard of MPs to animals, it is necessary to conduct accurate qualitative and quantitative analysis of MPs pollution in *R. bieti*. Based on the long-term field observation of *R. bieti* and the full understanding of their behavioral, the main objectives of this study were to explore (1) MPs ingestion in *R. bieti*; (2) The association between MPs pollution and food provisioning and tourism disturbance. (3) Does the strict dominance hierarchy of *R. bieti* increase MPs pollution by occupying high-quality artificial food resources? And (4) effects of MPs on gut microbiome diversity. These scientific questions can further understand the transport of MPs in rare and endangered animals, better explore the hazard of human activities to rare and endangered animals, and provide scientific basis for formulating effective protection measures to promote the effective protection of *R. bieti*.

## 2 Materials and methods

### 2.1 Study species and study location

The endangered Yunnan snub-nosed monkey (*Rhinopithecus bieti*) primarily inhabits dark coniferous forests and lives at the highest altitudes (2,600–4,700 m) of any nonhuman primate (Long et al., 1994). Its present geographic range is limited to the narrow strip between the upper Yangtze River/Jinshajiang and upper Mekong River/Lancangjiang in southwest China (Xia et al., 2020). The study area is located in the southern Hengduan Mountains, one of thirty-six global biodiversity hotspots. Inaccessible and sparsely populated, the *R. bieti*’s habitat is largely protected by nature reserves. The *R. bieti* is an arboreal herbivorous primate, feeding mainly on lichens (which are themselves an indicator of environmental quality). Previous observations have shown that *R. bieti* also spend a substantial amount of time on the ground. There, they forage for food such as bamboo shoots and herbs as well as displaying other unusual foraging behaviors (eating soil, bird eggs/birds, and insects in rotting trees). Wild *R. bieti* fear people and flee when approached more closely than 200 m.

**Figure 1:**
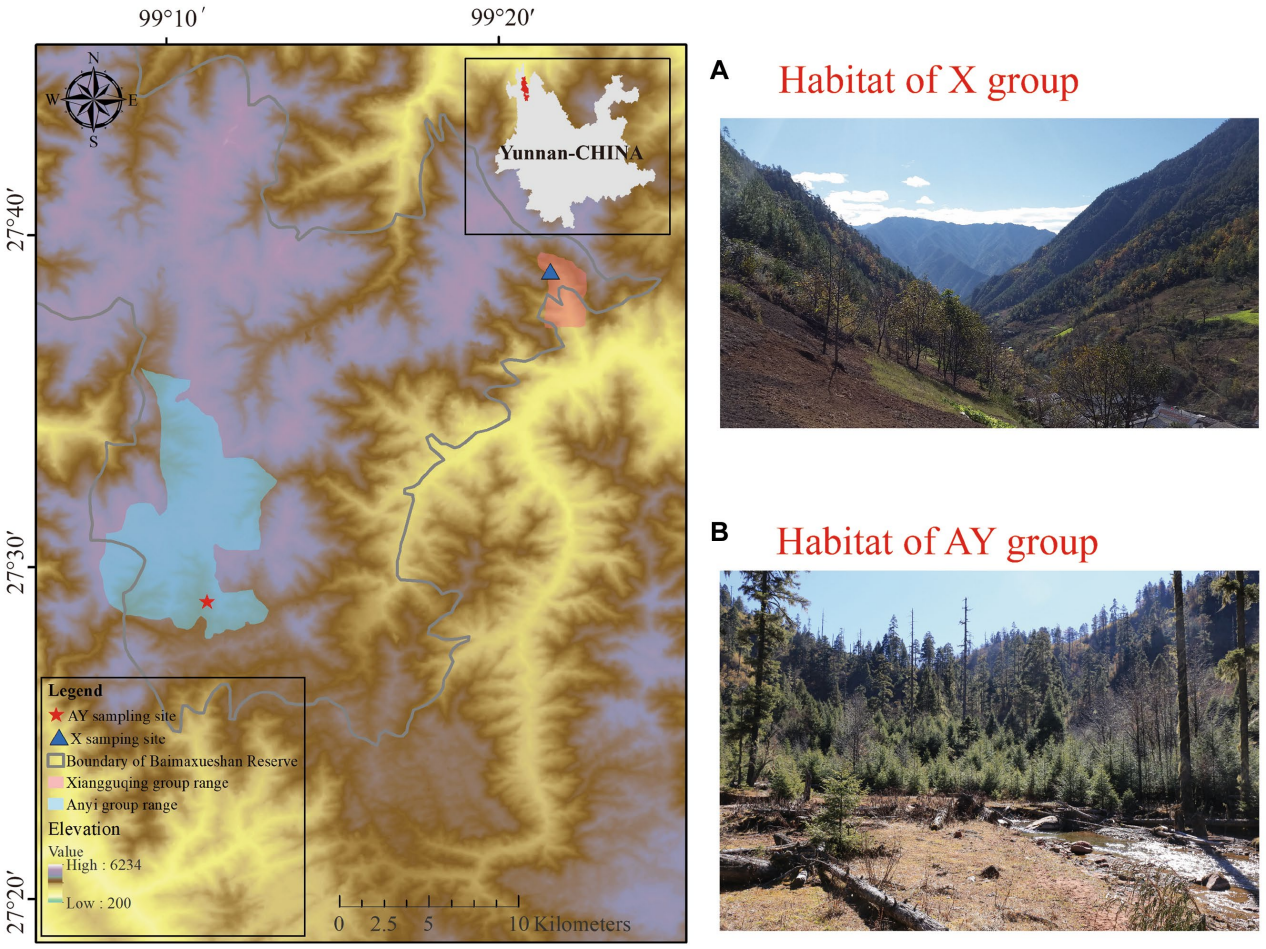
Study site and sampling site habitat.

Our research was carried out at two sites, Xiangguqing (Group X) and Anyi (Group AY), on the southern slopes of Baimaxueshan National Nature Reserve, Yunnan Province, China. Group X is a wild, but provisioned group which was separated from a larger wild group in 2008 and habituated in the Xiangguqing area (Ren et al., 2012; Xia et al., 2022c). Most of Group X’s food comes from the wild, but supplemental foods are provided regularly to meet the needs of scientific research and ecological tourism (Xia et al., 2022a). Foods including lichens, carrots, apples, peanuts, and lacquer tree fruits are provided twice daily (around 9:00 and 17:00 h) by reserve staff (Xia et al., 2022a). Although Group X is provisioned group and has had repeated exposure to tourism, tourists are not permitted to feed the monkeys, and the monkeys do not approach them for food. In general, the monkeys of Group X are not friendly to visitors and rarely initiate contact with them (Xia et al., 2016). In contrast, Group AY is a completely unhabituated wild group living 34 km away from Group X. Undisturbed by tourism, Group AY eats a natural diet and is even more fearful of people than Group X (Xia et al., 2022a) (Figure 1).

### 2.2 Sample collection

As residual portions of unabsorbed food, feces samples can provide direct evidence that animals have ingested MPs (Liu M. et al., 2023). From April 17 to April 26, 2023, a total of 40 fresh feces samples were collected, including 20 samples from Group X and 20 samples from Group AY. 15 July 2023 to 20 July 2023, we collected three wild food samples and three provisioning food samples from group X. On August 15, 2023, a large debris flow occurred in the area of group X, the road was washed out, and tourism activities were forced to close. We collected another 45 samples of group X from November 18 to 28, 2023, to verify the MPs of the provided group without tourism activities, which was named Group C. The feces sampling process was similar to the methodology used in our previous study of the gut microbiomes of *R. bieti* (Xia et al., 2022a,c). All samples were stored in plastic-free glass dishes, sealed, and immediately transferred to the freezer at −80°C after labeling. Chemical analysis to detect MPs was performed within 1 week after sample collection.

### 2.3 Sample processing procedures

Before processing, removed the large and difficult to digest particles in feces. In order to prevent plastic contamination after collection, we strictly observed a plastic-free principle during the experiment. First, we dried the sample at 60°C, and then we rinsed all glassware and instruments with deionized water to prepare them. Eighty five feces samples (weight 0.920 ~ 4.532 g, Supplementary Table S1) and six food sample were tested concentrated sulfuric acid (68%) three times the volume of the feces was added to the sample, allowed to sit for 24 h, then heated to 60°C for 3 h to dissolve the protein. The large particles were filtered out with a 500 μm large-aperture filter screen, followed by vacuum filtration. After rinsing with ultra-pure water and ethanol three times, the filter membrane was immersed in an ethanol solution. Thirty minutes of ultrasonic treatment dispersed the particles on the filter membrane into the ethanol solution. The filter membrane was then removed from the ethanol solution and rinsed three more times with additional ethanol to wash any remaining particles back into the solution. Finally, the ethanol solution was concentrated and its droplets placed on highly reflective glass. Laser infrared imaging spectrometer (LDIR) testing was performed after the ethanol was completely volatilized. We used the Agilent 8700 LDIR to identify the abundance and size distribution of microplastics in feces samples. The automatic particle analysis method compared the samples with the Microplastics Starter 2.0 spectrum library (De Frond et al., 2021) to define the MPs (matching degree >0.65, particle size range: 20 ~ 500 μm). All samples were tested by the Shanghai WEIPU Testing Technology Group Co., LTD.

### 2.4 Dominance hierarchy

From October 15, 2023 to December 24, 2023, the interactive behaviors of Yunnan snub-nose monkeys were observed and recorded using the focal animal sampling and all-occurrence recording method (Altmann, 1974). Threatening, supplanting, chasing, fighting, and biting were classified as winning interactions, and avoiding, crouching and retreating were classified as losing interactions.

We used the Normalized David’s score (NDS) calculation method to calculate dominance among OMUs (de Vries et al., 2006), and the calculation method is as follows:

NDS={DS+N(N−1)/2}/N .

$\mathrm{DS}=\left(w+w_{2}\right)-\left(l+l_{2}\right)$ .

Where *N* is group size, *w* = ∑*D*_ij_ the sum of all *D*_ij_ for cow *i*; *l* = the sum of all *D*_ji_ values for cow *j*; *w*_2_ = *w* weighted by the w of interaction partners; *l*_2_ is *l* weighted by the *l* of interaction partners.

$D_{\mathrm{ij}}=P_{\mathrm{ij}}-\left(P_{\mathrm{ij}}-0.5\right)/\left(n_{\mathrm{ij}}+1\right);D_{\mathrm{ji}}=1-D_{\mathrm{ij}}$ .

*P*_ij_ = the proportion of interactions won by individual *i* against *j*; *n*_ij_ = the number of interactions between *i* and *j*.

The NDS determines the dominance hierarchies of an individual, the larger the NDS, the higher the dominance.

### 2.5 DNA extraction, PCR amplification, 16S rRNA gene amplicon sequencing and analysis of the gut microbiome

DNA was extracted using the MagaBio Soil/Feces Genomic DNA Purification Kit (Bioer Technology, Hangzhou, China) according to the manufacturer’s protocol. The purity and concentration of the DNA are assessed using a Nanodrop One (Thermo Fisher Scientific, MA, United States). The integrity of the nucleic acids was determined visually by electrophoresis on a 1.0% agarose gel containing ethidium bromide. PCR amplification of the V3-V4 hypervariable regions of the bacterial 16S rRNA gene was carried out in a 25 μL reaction using universal primer pairs (338F:5′-ACTCCTACGGGAGGCAGCA-3′ and 806R:5′-GGACTACHVGGGTWTCTAAT-3′). The PCR reaction (30 mL total volume) contained 15 μL2 × Gflex PCR Buffer, 0.6 μL Tks Gflex DNA Polymerase (1.25 U/μl), 1 μL (5 pmol/μl) forward primer, 1 μL (5 pmol/μl) reverse primer, template DNA (50 ng), and 12.4 μL PCR grade water. PCR amplification program was established according to the following procedure:94°C for 5 min, followed by 30 cycles of 94°C for 30 s, 56°C for 30 s, and 72°C for 20 s, and a final extension at 68°C for 5 min. Following concentration comparison of PCR products using GeneTools Analysis Software (Version 4.03.05.0, SynGene), the required volume for each sample is calculated based on the principle of equal mass, after which the PCR products are mixed. Library construction is carried out following the standard protocol of the ALFA-SEQ DNA Library Prep Kit, and the size of the library fragments is evaluated on the Qsep400 High-Throughput Nucleic Acid & Protein Analysis System (Hangzhou Houze Biotechnology Co., Ltd., China).

Paired-end reads were preprocessed using Trimmomatic software (Bolger et al., 2014) to detect and remove ambiguous bases (N) and discard low-quality sequences with an average quality score below 20. After trimming, clean paired-end reads were assembled using FLASH software (Reyon et al., 2012). Parameters of the assembly were: 10 bp of minimal overlapping, 200 bp of maximum overlapping, and 20% of maximum mismatch rate. Then, additional sequences were denoising using QIIME software (version 1.9.0) (Caporaso et al., 2010). Potential chimeras were detected and removed using VSEARCH (Rognes et al., 2016). Primer sequences were removed by Vsearch software and clustered to generate operational taxonomic units (OTU) with a similarity cut-off value of 97%(Rognes et al., 2016). A representative read of each OTU was selected using QIIME package (Caporaso et al., 2010). All representative reads were annotated and blasted against the SILVA reference database (release 128).^1^

### 2.6 Data analysis

Since different feces samples vary in weight, we standardized the sample by “content = number of MPs/g; relative abundance = the proportion of a particular MP in the total MPs in the sample.” MPs data were visually inspected using Q–Q plots and tested for normality, with non-parametric methods used to calculate differences for non-normal distributions. PERMANOVA test using Bray-Curtis dissimilarities was used to test the significant difference among the Group X, Group AY and Group C. Kruskal Wallis test was used to test the difference of MPs content among Group X, Group AY and Group C. The PCoA plot was constructed using Bray-Curtis distances to assess dissimilarity among these three groups (Gao et al., 2019). Independent *t*-test was used to test significant difference among different age sex and unit. Pearson correlation test was used to examine the association between the dominance hierarchies of social units and MPs ingestion. All the graphing were performed using OmicShare, a free online platform for data analysis.^2^ All data analyses were carried out using “base” RStudio version 3.5.1, and the significance threshold for all tests was *p* ≤ 0.05.

## 3 Results and discussion

### 3.1 Yunnan snub-nosed monkey exposure to microplastics

**Figure 2:**
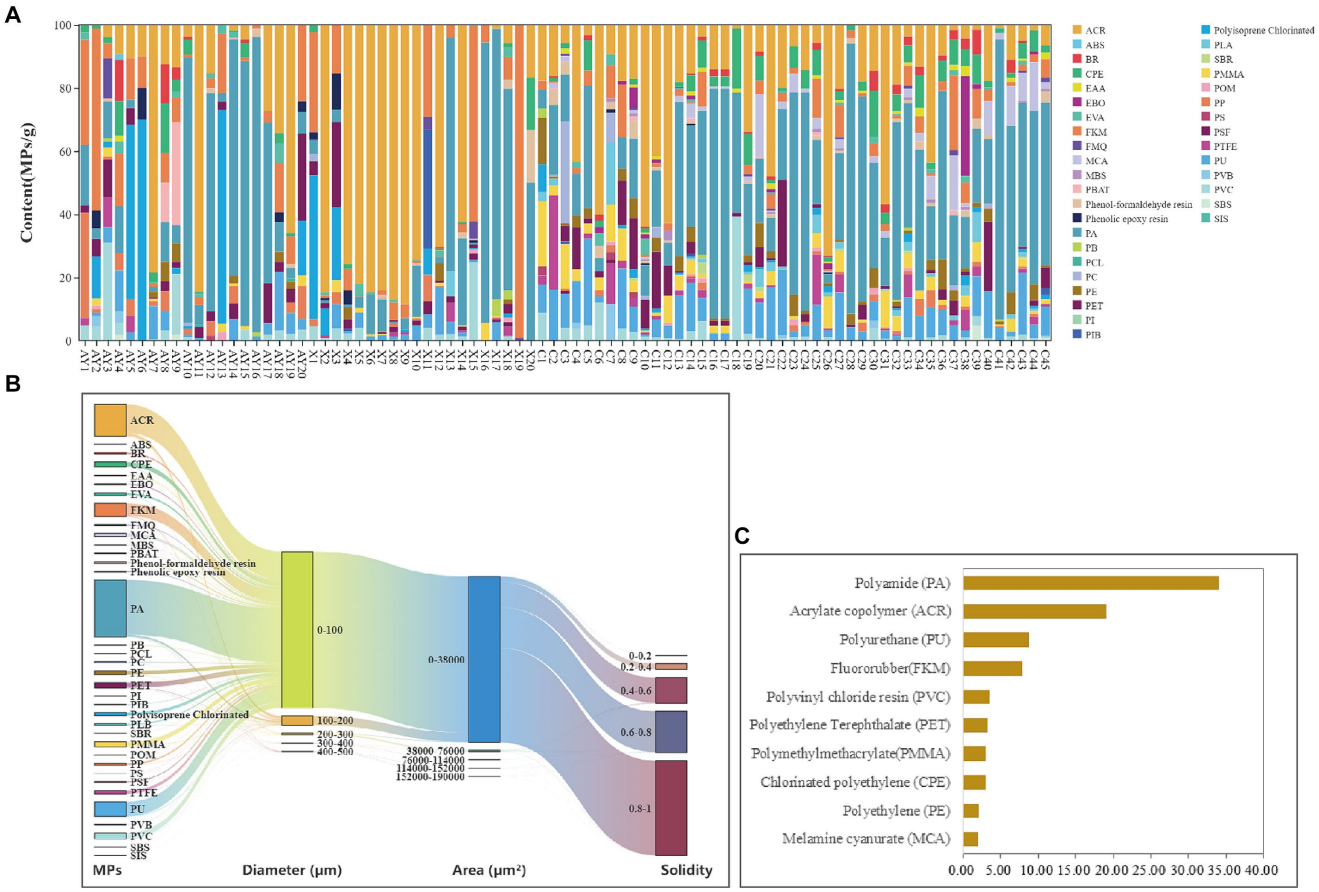
The MPs community and content in the Yunnan snub-nosed monkeys (*Rhinopithecus bieti*). **(A)** Relative abundance (%) of MPs. **(B)** Top 10 MPs distributions in feces samples. **(C)** The interrelationship among physicochemical properties of MPs in Yunnan snub-nosed monkeys. ABS, Acrylonitrile Butadiene Styrene; PI, Polyimide; SIS, Styrene isoprene styrene triblock copolymer; PCL, Polycaprolactone; SBS, Styrene butadiene styrene, PS, Polystyrene; SBR, Polymerized styrene butadiene rubber; MBS, Methyl methacrylate butadiene styrene; PIB, Polyisobutylene; POM, Polyoxymethylene; PB, Polybutadiene; PVB, Polyvinyl butyral; PBAT, Polybutylene adipate terephthalate; EAA, Ethylene acrylic acid; FMQ, Fluorosilicone rubber; PSF, Polysulfone; EBO, Ethylene bis oleamide; PC, Polycarbonate; BR, Butadiene rubber; PLA, Polylactic acid; PP, Polypropylene; EVA, Ethylene vinyl acetate copolymer; PTFE, Polytetrafluoroethylene; MCA, Melamine cyanurate; PE, Polyethylene; CPE, Chlorinated polyethylene; PMMA, Poly methyl meth acrylate; PET, Polyethylene terephthalate; PVC, Polyvinyl chloride resin; FKM, Fluororubber; PU, Polyurethane; ACR, Acrylate copolymer; PA, Polyamide.

Microplastics were found in all 85 feces samples at ranging from 7.743 ~ 222.898 MPs/g (average 75.263 ± 58.141), and these MPs belong to 36 plastic types (see Figure 2A). Our study is the first to demonstrate the ingestion of MPs in Yunnan snub-nosed monkeys using feces samples. Although it is not surprising that MPs are widespread in terrestrial ecosystems. These results are surprising given that the monkeys inhabit high-altitude old growth forests with little to no human settlement. For the Tibetan Plateau with unique geographical unit characteristics and climatic conditions (Liu X. et al., 2023), MPs can be transported to remote, uninhabited areas such as the wind, rain (Amato-Loureno et al., 2021) or glacier melting (Talukdar et al., 2023), polluting air, water and food within the habitat, thus formed the “atmospheric deposition—vegetation—animals” MPs transport model (Liu X. et al., 2023). At the same time, “atmospheric deposition-snow melt and fresh water flow” is also one of the important ways for the transport model of MPs on the Qinghai-Tibet Plateau. For example, rubber, PA, PP, PET, and PE were all detected in the snow water in Tibetan Plateau (Zhang et al., 2021).

The average content of MPs in the feces of Yunnan snub-nosed monkey is lower than that of Tibetan Wild Ass (*Equus Kiang*), but the types of MPs (36 types) were higher than that of *Equus Kiang* (29 types) (Liu X. et al., 2023). The MPs ingestion in Yunnan snub-nosed monkeys was relatively high. Therefore, the long-term effects of these microplastics on terrestrial wildlife in the area of the Sanjiang River parallel flow deserve further investigation. The dominant MPs included Fluororubber (FKM, 7.81%), Polyurethane (PU, 8.75%), Acrylate copolymer (ACR, 19.07%), and Polyamide (PA, 33.97%) (Figure 2C).

Previous studies have demonstrated the prevalence of MPs in air and fresh water environments and found the PP, PE, PS, and PET are the main polymer components of MPs in atmospheric (Chen et al., 2019). Lightweight MPs such as PE, PS, and PP are the main components of MPs in freshwater (Bhardwaj et al., 2024). Our results showed that the predominant types of MPs in the feces of Yunnan snub-nosed monkey were not consistent with those in the air and freshwater. The main MPs in the feces of Yunnan snub-nosed monkey may be related to the production and living of local residents, such as textiles, coatings, adhesives and personal care products, and related PA, ACR, PU, and FKM. We hypothesized that in addition to the “atmospheric deposition—vegetation—animals” and “atmospheric deposition-snow melt and fresh water flow” transport model (Liu X. et al., 2023), human activities (including tourism) should also be an important link in the transport chain of MPs in Yunnan snub-nosed monkeys.

The diameter < 100 μm size category was the most abundant, accounting for 93.28% of the total MPs, the 0–38,000 um^2^ area category were the most abundant, accounting for 98.95%, and the 0.8–1 solidity category were the most abundant, accounting for 56.46%. Overall, the majority of MPs in the feces of Yunnan snub-nosed monkeys were characterized by a diameter of less than 100 um, an area of 0–38,000 um, and a solidity of 0.8–1 (Figure 2B). The size of MPs can affect the ingestion of MPs by organisms. The smaller the size of MPs, the higher the probability of MPs being eaten by organisms (Bhardwaj et al., 2024). Experiments on zebrafish showed that the smaller the particle size of MPs, the more serious the damage to the visceral tissue. Therefore, the impacts of these MPs on Yunnan snub-nosed monkeys and ecosystems in the three rivers parallel flow region should be taken seriously.

### 3.2 Effects of food provisioning and tourism on feces microplastics in Yunnan snub-nosed monkey

**Figure 3:**
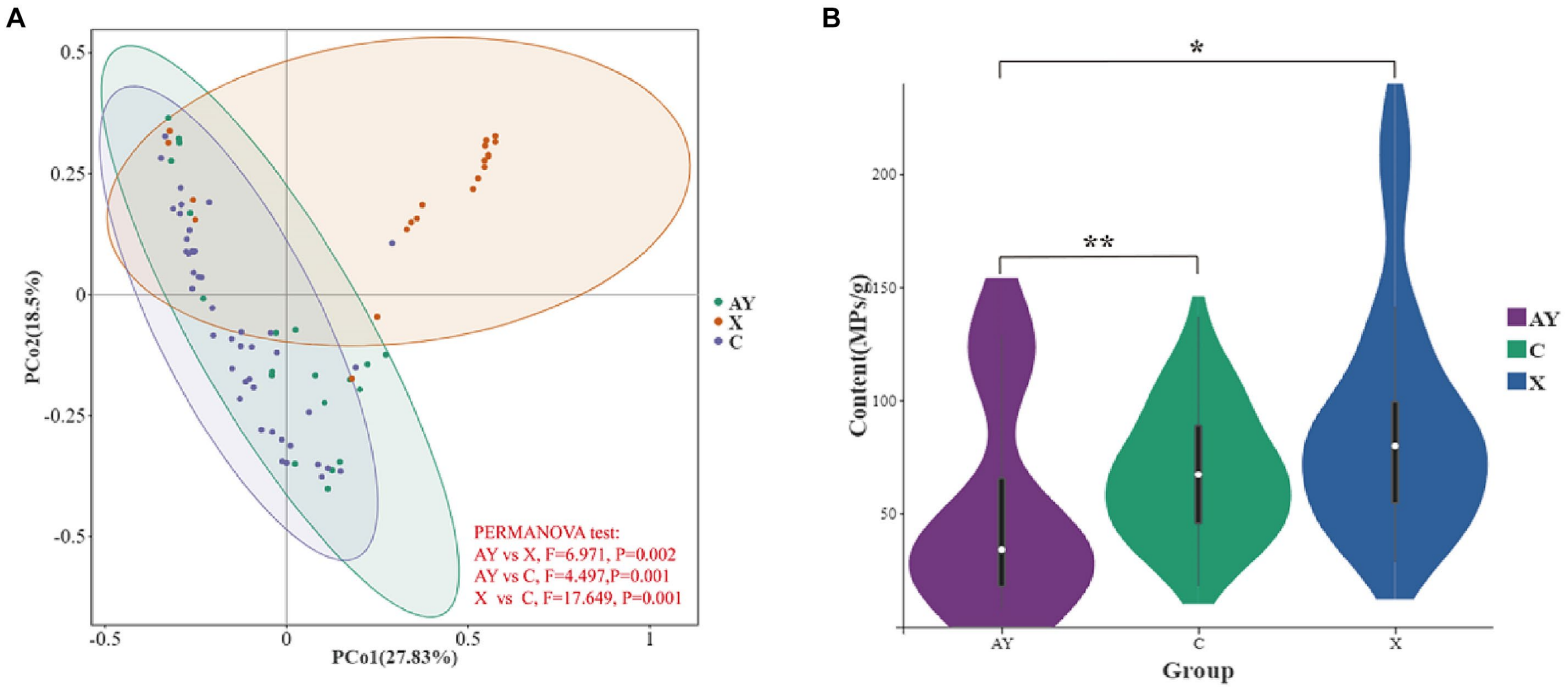
Effects of food provisioning and tourism on feces microplastics in Yunnan snub-nosed monkey. **(A)** Relative abundance of MPs. **(B)** Content of MPs. *p< 0.05, **p< 0.01.

We found significant dissimilarity in the MPs relative abundance among the wild foraging (AY), tourism and food provisioned (X), food provisioned (C) of Yunnan snub-nosed monkey (Figure 3A, PERMANOVA test, *F* = 23.922, *p* < 0.001). The content of MPs in group AY (51.78 ± 45.68MPs/g, *n* = 20) was significantly lower than that in group X (89.50 ± 50.48, *n* = 20, Kruskal Wallis test, *p* = 0.006; Figure 3B), indicating that food provisioning and tourism significantly increased the content of MPs in the feces of Yunnan snub-nosed monkeys. The content of MPs in group C (67.47 ± 28.19, *n* = 45) was significantly higher than that in AY group (Kruskal Wallis test, *p* = 0.017, Figure 3B), and slightly lower than that in X group, but the difference was not significant (Kruskal Wallis test, *p* = 0.125, Figure 3B). In conclusion, food provisioning increased the exposure of MPs in Yunnan snub-nosed monkeys, and the disturbance caused by food provisioning was greater than that caused by tourism.

Excluding the disturbances of food provisioning and tourism, there is little difference between the atmosphere, water, and other anthropogenic disturbances experienced by Group AY and Group X, living within 35 km of each other. Under the dual influence of food provisioning and tourism, MPs in Yunnan snub-nosed monkey feces did increase. It has been confirmed that tourism activities lead to an increase in microplastics (Gao et al., 2021), and MPs in the Qinghai-Tibet Plateau have also been confirmed to be related to tourism (Feng et al., 2021). Provisioning food (lichens, carrots, apples, peanuts, and lacquer tree fruits) is packed and transported with plastic. Meanwhile, we found that the content of MPs in artificial food (195.783 ± 60.372, *n* = 3) is significantly higher than that in wild food (30.001 ± 12.147, *n* = 3; Kruskal Wallis test, *p* < 0.001). However, our results found that tourism alone (Group X vs. Group C) did not significantly increase the content of MPs as food provisioning. There are several measures in place to protect the monkeys may have reduced MPs pollution from tourist activities, such as limiting the number of visitors (on average just under 74 people per day between 2022.5.28 and 2023.2.10) and the times when they can visit (morning only), forbidding physical contact between humans and monkeys, prohibiting tourists from feeding monkeys, and regular garbage removal.

### 3.3 The composition and content of MPs in different age, sex and social units

**Figure 4:**
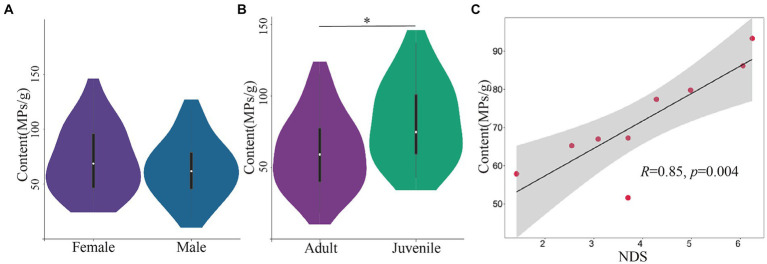
The composition and content of MPs in different age, sex and social units. **(A)** Content of MPs between different sex groups. **(B)** Content of MPs between different age groups. **(C)** Dominance hierarchies correlated with content of MPs. *p<0.05.

The analysis of feces samples from 45 individuals in group C showed that there was no significant difference in the content of MPs between different sex groups (female, 71.27 ± 29.52, *n* = 24; male, 63.13 ± 26.62, *n* = 21; independent *T* test, *t* = 0.966, *p* = 0.339, Figure 4A). However, the feces MPs content of adult Yunnan snub-nosed monkeys (60.19 ± 25.71, *n* = 30) was significantly lower than that of juvenile (82.04 ± 28.05, *n* = 15; independent-samples *T* test, *t* = 2.607, *p* = 0.012, Figure 4B). Juvenile monkeys showed more affiliative behaviors, rather than hostile behaviors toward visitors and staff than adult monkeys (Xia et al., 2016). Juvenile individuals tend to be more playful and exploratory than adults (Li et al., 2014). Juvenile monkeys have also been observed repeatedly playing with backpacks of tourists and staff and licking plastic food packaging. Active interaction with humans may be one of the reasons for the increase of MPs in juvenile feces.

There was no significant difference in MPs content (PERMANOVA test, *F* = 1.11, *p* = 0.27) and relative abundance (PERMANOVA test, *F* = 1.25, *p* = 0.17) among OMUs. But the social dominance hierarchies among OMUs (for the dominance hierarchies data, see Supplementary Table S2) was positively correlated with the exposure of MPs (Pearson correlation coefficient, *R*^2^ = 0.846, *p* = 0.004; Figure 4C). Compared with low-energy wild food, high-energy artificial food full of MPs is a precious resource, and the possession of precious resources is directly related to dominance hierarchies. Previous research also showed that the high dominance hierarchies individual or unit preferentially occupy the precious resources (Tibbetts et al., 2022), which leads to high MPs ingestion.

### 3.4 Effects of MPs on gut microbiome diversity

**Figure 5:**
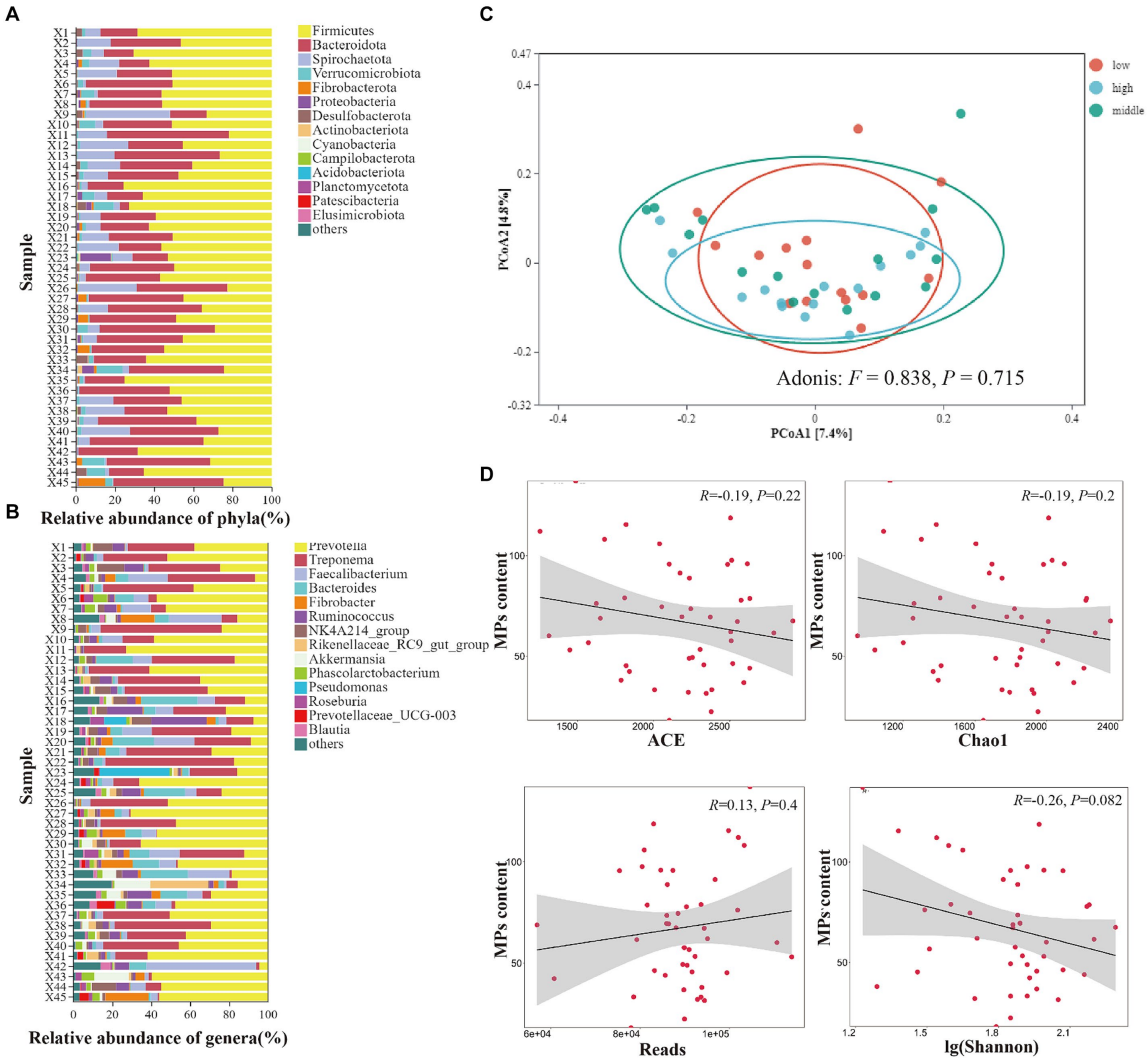
The effects of microplastics on gut microbiomes of Yunnan snub-nosed monkey. **(A)** Relative abundance of phyla(%), **(B)** Relative abundance of genera(%). **(C)** The PCoA analysis of gut microbiome community among different MPs content groups. **(D)** Relationship between MPs content and gut microbes diversity.

We collected 45 feces samples from 45 individuals belonging to three MPs content groups (high, medium and low groups, 15 samples each group). The dominant phyla included Firmicutes, Bacteroidota, Spirochaetota, Verrucomicrobiota, Fibrobacterota (Figure 5A). The dominant genera included Prevotella, Treponema, Faecalibacterium, Bacteroides, Fibrobacter (Figure 5B). With the increase of MPs content, the alpha diversity (ACE, chao1, reads, simpson, shannon_10) of gut microbes were not significantly affected (Figure 5D). Furthermore, the PCoA analysis, using Bray-curtis distance, displayed a mixed pattern (Figure 5C), with no significant difference in the gut microbiome community among different MPs content groups (Adonis: *F* = 0.838, *p* = 0.715), indicating MPs content did not affect the beta diversity of gut microorganisms in Yunnan snub-nosed monkeys.

Our results were different from most research that MPs can cause gut microbiome dysbiosis (Zhu et al., 2018; Fackelmann and Sommer, 2019; Ju et al., 2019). One of the main reasons may be the real-life effects of microplastics concentration on the gut microbiota of wild animals remains a mystery (Fackelmann and Sommer, 2019), in general, the difference in MPs content between the control group and the experimental group was very large (Lu et al., 2018; Zhu et al., 2018; Ju et al., 2019; Qiao et al., 2019), while the MPs content of the wild Yunnan snub-nosed monkey in our study did not show such a large gradient difference. In addition, MPs was found to have significant effects on gut microbiota of almost all small and medium-sized species [e.g., *Folsomia candida* (Ju et al., 2019), Zebrafish (Jin et al., 2018), and mice (Lu et al., 2018)]. Is body-mass a determinant? In the seabird study, there was no significant association between MPs abundance and gut microbiome diversity without body-mass normalization (Fackelmann et al., 2023). Species with different body sizes may have different levels of tolerance to the same dose of MPs (currently only a hypothesis). Social contact leads to the convergence of gut microbiome (Moeller et al., 2016; Xia et al., 2022c). Yunnan snub-nosed monkeys are social animals, and individuals transfer among social units (Xia et al., 2022b), contact behaviors such as grooming among individuals make the high similarity in the gut microbiome among the individuals (Xia et al., 2022c). These behavioral rules may be one of the reasons for reducing the effect of MPs on gut microbiome dysbiosis in Yunnan snub-nosed monkeys.

## 4 Conclusion

In summary, the present study provides the first evidence of MPs ingestion by Yunnan snub-nosed monkeys, and a total of 36 types of MPs with an average of 75.263 ± 58.141MPs/g. Human activities (food provisioning and tourism) significantly increased the content of MPs, but tourism alone did not significantly increase the content of MPs like food provisioning. Affiliative, playful and exploratory behaviors of juvenile may lead to higher MPs ingestion, while MPs ingestion did not different by sex. The social dominance hierarchies among OMU was positively correlated with the MPs ingestion. The current level of MPs pollution did not cause gut microbiome dysbiosis of Yunnan snub-nosed monkeys. Our study researched the MPs ingestion and gut microbiome dysbiosis of Yunnan snub-nose monkeys from the behavioral and ecological and gut microbiome perspectives, and provided scientific basis for formulating effective protection measures (replace plastic with non-plastic products) and promoting the effective protection of rare and endangered animals.

## Data availability statement

The original contributions presented in the study are publicly available. This data can be found here: https://www.ncbi.nlm.nih.gov/bioproject/, accession number PRJNA1144169.

## Ethics statement

The animal study was approved by China West Normal University Research Ethics Committee. The study was conducted in accordance with the local legislation and institutional requirements.

## Author contributions

WX: Conceptualization, Data curation, Funding acquisition, Investigation, Methodology, Project administration, Resources, Software, Validation, Visualization, Writing – original draft, Writing – review & editing. JZ: Data curation, Formal analysis, Investigation, Methodology, Software, Writing – original draft. ZL: Investigation, Methodology, Software, Writing – original draft. LL: Data curation, Investigation, Methodology, Software, Writing – original draft. YZ: Data curation, Formal analysis, Investigation, Methodology, Software, Writing – original draft. SF: Data curation, Formal analysis, Investigation, Methodology, Software, Writing – original draft. AK: Conceptualization, Writing – review & editing. DL: Conceptualization, Funding acquisition, Methodology, Project administration, Resources, Supervision, Writing – review & editing.

## Footnotes

^1^http://www.arb-silva.de/

^2^https://www.omicshare.com/tools
