# Supplementary material for: Microplastics and gut microbiomes impact on Yunnan snub-nosed monkeys in the Three Parallel Rivers region in China
Source: Front Microbiol. 2024 Aug 12;15:1449522. doi: 10.3389/fmicb.2024.1449522 (PMC11346417; doi:10.3389/fmicb.2024.1449522)
Supplement: Supplementary file 1 [file Table_1.DOCX]

**Table S1 Sample information table**

| **Sample ID** | **Sampling time** | **OMU** | **Age** | **Sex** | **Original sample weight (g)** | **Test sample weight/Dry weight of sample (g)** | **Microplastic particle** |
| --- | --- | --- | --- | --- | --- | --- | --- |
| AY1 | 2023-4-20 | unknown | unknown | unknown | 2.807 | 1.292 | 44 |
| AY2 | 2023-4-20 | unknown | unknown | unknown | 2.626 | 0.584 | 19 |
| AY3 | 2023-4-20 | unknown | unknown | unknown | 3.422 | 2.864 | 26 |
| AY4 | 2023-4-23 | unknown | unknown | unknown | 2.973 | 1.065 | 33 |
| AY5 | 2023-4-23 | unknown | unknown | unknown | 2.978 | 1.727 | 252 |
| AY6 | 2023-4-23 | unknown | unknown | unknown | 1.102 | 0.818 | 96 |
| AY7 | 2023-4-23 | unknown | unknown | unknown | 2.955 | 1.721 | 80 |
| AY8 | 2023-4-23 | unknown | unknown | unknown | 1.625 | 1.247 | 32 |
| AY9 | 2023-4-23 | unknown | unknown | unknown | 2.966 | 1.162 | 14 |
| AY10 | 2023-4-23 | unknown | unknown | unknown | 3.039 | 1.468 | 71 |
| AY11 | 2023-4-23 | unknown | unknown | unknown | 1.526 | 1.526 | 24 |
| AY12 | 2023-4-23 | unknown | unknown | unknown | 3.043 | 1.351 | 173 |
| AY13 | 2023-4-23 | unknown | unknown | unknown | 1.685 | 1.331 | 50 |
| AY14 | 2023-4-23 | unknown | unknown | unknown | 2.952 | 2.078 | 61 |
| AY15 | 2023-4-23 | unknown | unknown | unknown | 2.992 | 1.097 | 239 |
| AY16 | 2023-4-23 | unknown | unknown | unknown | 3.032 | 2.325 | 18 |
| AY17 | 2023-4-23 | unknown | unknown | unknown | 4.532 | 4.532 | 154 |
| AY18 | 2023-4-23 | unknown | unknown | unknown | 3.035 | 1.610 | 69 |
| AY19 | 2023-4-23 | unknown | unknown | unknown | 2.906 | 1.123 | 131 |
| AY20 | 2023-4-23 | unknown | unknown | unknown | 1.806 | 0.688 | 6 |
| X1 | 2023-4-17 | unknown | sub-adult | F | 2.867 | 0.565 | 42 |
| X2 | 2023-4-17 | unknown | adult | M | 2.626 | 1.026 | 90 |
| X3 | 2023-4-17 | unknown | adult | M | 3.422 | 0.822 | 103 |
| X4 | 2023-4-20 | unknown | sub-adult | M | 2.976 | 0.976 | 54 |
| X5 | 2023-4-20 | unknown | juvenile | M | 3.041 | 0.557 | 38 |
| X6 | 2023-4-20 | unknown | sub-adult | M | 2.991 | 0.351 | 10 |
| X7 | 2023-4-23 | unknown | sub-adult | M | 3.032 | 1.354 | 264 |
| X8 | 2023-4-23 | unknown | adult | F | 2.978 | 0.771 | 32 |
| X9 | 2023-4-24 | unknown | adult | F | 3.000 | 0.988 | 52 |
| X10 | 2023-4-24 | unknown | adult | F | 3.006 | 1.145 | 107 |
| X11 | 2023-4-24 | unknown | adult | F | 2.252 | 0.993 | 116 |
| X12 | 2023-4-24 | unknown | adult | F | 3.008 | 0.708 | 65 |
| X13 | 2023-4-24 | unknown | adult | F | 2.674 | 0.812 | 37 |
| X14 | 2023-4-26 | unknown | sub-adult | M | 2.560 | 0.987 | 220 |
| X15 | 2023-4-26 | unknown | adult | F | 2.663 | 1.093 | 87 |
| X16 | 2023-4-26 | unknown | juvenile | F | 2.968 | 0.853 | 78 |
| X17 | 2023-4-26 | unknown | adult | M | 3.013 | 0.689 | 55 |
| X18 | 2023-4-26 | unknown | adult | F | 3.004 | 0.542 | 32 |
| X19 | 2023-4-26 | unknown | juvenile | F | 3.025 | 0.644 | 91 |
| X20 | 2023-4-26 | unknown | adult | M | 3.026 | 0.728 | 29 |
| C1 | 2023-11-18 | QiGe | adult | M | 3.339 | 1.882 | 34 |
| C2 | 2023-11-18 | QiGe | juvenile | M | 1.710 | 1.710 | 185 |
| C3 | 2023-11-18 | QiGe | adult | F | 2.743 | 1.743 | 121 |
| C4 | 2023-11-18 | AMU | adult | M | 3.093 | 1.684 | 53 |
| C5 | 2023-11-18 | ErJi | adult | F | 2.862 | 1.862 | 62 |
| C6 | 2023-11-18 | ErJi | juvenile | M | 1.826 | 1.022 | 50 |
| C7 | 2023-11-18 | ErJi | adult | F | 3.180 | 1.883 | 86 |
| C8 | 2023-11-19 | ErJi | adult | F | 2.566 | 1.601 | 79 |
| C9 | 2023-11-19 | ErJi | adult | M | 3.457 | 1.457 | 111 |
| C10 | 2023-11-19 | ErJi | adult | F | 2.910 | 2.690 | 155 |
| C11 | 2023-11-19 | AMU | adult | M | 3.222 | 2.339 | 89 |
| C12 | 2023-11-19 | AMU | adult | M | 2.982 | 1.419 | 126 |
| C13 | 2023-11-19 | Ershida | adult | F | 3.063 | 1.810 | 209 |
| C14 | 2023-11-19 | ShuaiGe | adult | F | 3.033 | 2.433 | 233 |
| C15 | 2023-11-20 | ShuaiGe | adult | M | 3.131 | 1.520 | 81 |
| C16 | 2023-11-20 | HongDian | adult | M | 3.086 | 1.627 | 128 |
| C17 | 2023-11-20 | ShuaiGe | adult | F | 2.137 | 1.896 | 128 |
| C18 | 2023-11-20 | HongDian | juvenile | M | 1.583 | 1.583 | 84 |
| C19 | 2023-11-20 | ShuaiGe | juvenile | M | 2.391 | 1.391 | 133 |
| C20 | 2023-11-20 | HongDian | juvenile | M | 2.985 | 2.308 | 142 |
| C21 | 2023-11-21 | HongDian | juvenile | M | 2.944 | 1.491 | 177 |
| C22 | 2023-11-21 | AMU | adult | M | 3.230 | 1.743 | 120 |
| C23 | 2023-11-21 | MiLi | adult | F | 2.913 | 1.713 | 103 |
| C24 | 2023-11-21 | LiuXin | juvenile | F | 0.920 | 0.920 | 84 |
| C25 | 2023-11-21 | HuoBiZi | adult | F | 3.205 | 1.900 | 88 |
| C26 | 2023-11-21 | HuoBiZi | juvenile | F | 1.517 | 1.517 | 208 |
| C27 | 2023-11-21 | HuoBiZi | adult | F | 3.063 | 2.224 | 138 |
| C28 | 2023-11-21 | ShuaiGe | juvenile | F | 1.583 | 1.583 | 118 |
| C29 | 2023-11-21 | HongDian | juvenile | M | 1.878 | 1.578 | 106 |
| C30 | 2023-11-21 | Ershida | adult | M | 2.679 | 1.061 | 48 |
| C31 | 2023-11-21 | Ershida | adult | F | 2.481 | 1.945 | 190 |
| C32 | 2023-11-21 | HongDian | juvenile | F | 2.033 | 2.033 | 195 |
| C33 | 2023-11-21 | HongDian | adult | F | 3.029 | 2.477 | 109 |
| C34 | 2023-11-22 | HongDian | adult | F | 2.970 | 1.144 | 38 |
| C35 | 2023-11-22 | HongDian | adult | F | 2.842 | 1.208 | 94 |
| C36 | 2023-11-22 | HongDian | juvenile | M | 3.050 | 1.464 | 62 |
| C37 | 2023-11-25 | MiLi | adult | M | 3.006 | 2.816 | 63 |
| C38 | 2023-11-25 | MiLi | adult | F | 2.500 | 2.500 | 92 |
| C39 | 2023-11-25 | ErJi | adult | F | 2.846 | 1.996 | 64 |
| C40 | 2023-11-25 | AMU | adult | M | 3.150 | 1.786 | 141 |
| C41 | 2023-11-26 | AMU | juvenile | F | 2.868 | 1.842 | 195 |
| C42 | 2023-11-26 | ErJi | adult | F | 2.891 | 1.035 | 72 |
| C43 | 2023-11-26 | AMU | juvenile | M | 2.786 | 2.786 | 158 |
| C44 | 2023-11-28 | HuoBiZi | juvenile | M | 1.829 | 1.604 | 118 |
| C45 | 2023-11-28 | MiLi | adult | F | 1.661 | 1.232 | 138 |

**Table S2 the social dominance hierarchies among OMUs**

|  | LiuXin | Ershida | HuoBiZi | ShuaiGe | HongDian | AMU | QiGe | MiLi | ErJi | w | w2 | w+w2 | l+l2 | DS | NDS |
| --- | --- | --- | --- | --- | --- | --- | --- | --- | --- | --- | --- | --- | --- | --- | --- |
| LiuXin |  | 0.65 | 0.893939 | 0.865854 | 0.759615 | 0.681818 | 0.793478 | 0.806667 | 0.790323 | 6.241694 | 23.04492 | 29.28661 | 9.111364 | 20.17525 | 6.241694 |
| Ershida | 0.35 |  | 0.76087 | 0.736364 | 0.914063 | 0.833333 | 0.768293 | 0.827273 | 0.863636 | 6.053831 | 21.2878 | 27.34163 | 8.85715 | 18.48448 | 6.053831 |
| HuoBiZi | 0.106061 | 0.23913 |  | 0.794643 | 0.865854 | 0.662162 | 0.693548 | 0.82 | 0.797872 | 4.97927 | 16.52006 | 21.49934 | 12.6859 | 8.813434 | 4.97927 |
| ShuaiGe | 0.134146 | 0.263636 | 0.205357 |  | 0.763889 | 0.525 | 0.847826 | 0.744444 | 0.795455 | 4.279754 | 13.86159 | 18.14134 | 15.62356 | 2.517784 | 4.279754 |
| HongDian | 0.240385 | 0.085938 | 0.134146 | 0.236111 |  | 0.785714 | 0.609375 | 0.815789 | 0.791667 | 3.699125 | 11.6832 | 15.38233 | 18.09021 | -2.70788 | 3.699125 |
| AMU | 0.318182 | 0.166667 | 0.337838 | 0.475 | 0.214286 |  | 0.775 | 0.560606 | 0.85 | 3.697578 | 12.52592 | 16.2235 | 18.94529 | -2.7218 | 3.697578 |
| QiGe | 0.206522 | 0.231707 | 0.306452 | 0.152174 | 0.390625 | 0.225 |  | 0.646341 | 0.928571 | 3.087392 | 10.10583 | 13.19322 | 21.40669 | -8.21347 | 3.087392 |
| MiLi | 0.193333 | 0.172727 | 0.18 | 0.255556 | 0.184211 | 0.439394 | 0.353659 |  | 0.766667 | 2.545546 | 8.725821 | 11.27137 | 24.36145 | -13.0901 | 2.545546 |
| ErJi | 0.209677 | 0.136364 | 0.202128 | 0.204545 | 0.208333 | 0.15 | 0.071429 | 0.233333 |  | 1.415809 | 6.155894 | 7.571703 | 30.82942 | -23.2577 | 1.415809 |
| l | 1.758306 | 1.946169 | 3.02073 | 3.720246 | 4.300875 | 4.302422 | 4.912608 | 5.454454 | 6.584191 |  |  |  |  |  |  |
| l2 | 7.353058 | 6.91098 | 9.665172 | 11.90331 | 13.78933 | 14.64287 | 16.49409 | 18.907 | 24.24523 |  |  |  |  |  |  |
